# The Philosophy of Medical Science, Boylston's Prize Essay, 1849

**Published:** 1853-07

**Authors:** 


### BIBLIOGRAPHICAL.

*The Philosophy of Medical Science, Boylston's Prize Essay,* 1849. By Dr. E. Leigh. Boston. Tickner, Reed & Fields.

Dr. Leigh has, in the little publication before us, attacked several of the positions assumed by Dr. Bartlett in his essay on the Philosophy of Medical Science. Dr. Bartlett is well known as an able thinker, a zealous student, a learned physician, and an elegant and exact writer. He leans decidedly towards the French school, and is a follower of Louis, not only in his system, as a pupil, but in his method of investigation, his earnest love of truth, and his prudent and wholesome scepticism.

In his work on Medical Philosophy, Dr. Bartlett has stoutly rejected all mere inferences not fully substantiated by facts, and has laid down certain laws, designed to check the exuberant fancy of theorists, and to keep young and enthusiastic votaries of science within proper limits. He sees, as every man of common sense and common observation must see, that nothing has so retarded the progress of medical science, as the prevalence of fashions in physic, the dissemination of certain systems and theories to fit which facts have been distorted in every possible manner. To obviate this difficulty in future and to sweep away all the rubbish with which these false notions have cumbered medical science, Dr. B. has laid down a rule, that all science consists in facts and their relationships.

Dr. Leigh takes exception to this and seeks to establish another definition of science. Escaped as the Dr. is, in common with his brethren from that Egyptian bondage of theory, in which men were required to construct bricks without straw, laboring for centuries in manufacturing theories without facts to make them from, he nevertheless shrinks from the barrenness of the sea to which the present leaders of medical science have brought him, and longs for the flesh-pots of Egypt again. He would go back to the good old way of speculating and reasoning upon his observations, and building theories as vast and idle and empty as the pyramids. He lays down as his "platform," to borrow a word from the politicians, three propositions; that science embraces, 1st. ***Primary truths,*** or fundamental principles upon which its reasonings are based; 2nd. ***Ascertained and classified facts;*** and 3d. ***General ideas, truths and principles*** arrived at from the facts.

As the question started by Dr. L. is one of some importance, since it involves the very mode of progress of a great science, we propose to pause a little to consider his propositions and to compare his system with that of the New York professors.

We confess that we were a little puzzled at first to comprehend what Dr. Leigh meant by his primary truths. We knew of no inspired volume of medicine which was to furnish future generations with a law to guide their mental operations. We thought that the primary truths of all science were, after all, ultimate facts, arrived at by close observation and much study. The Dr., however, explains himself by an explanation which we would be glad to have explained. He plunges into the dead sea of metaphysics, and brings up from its drowned cities of thought, some fragments inscribed with such hieroglyphics as "pure intellection" "intuitive perception" and other shibboleths of the Germano-Yankee transcendental school of mental philosophy. What on earth these notions, even supposing them to be proven, have to do with a philosophy of medical science, we cannot imagine. Such general principles as that "every effect has a cause," belong as much to shoemaking as to medical science, for most assuredly if Crispin were not convinced that his fingers and tools stood in the relation of cause to the desired effect—boots, the excellent cordwainer would cease to be, and mankind would go barefoot. The Dr. advances nothing more than a few principles which are postulates in every human action, axioms in every chain of reasoning and not at all peculiar to medicine or to any other science. Hence we feel as fully justified in setting aside the first division of his definition of science, as in skipping the first book of Mr. Brown's autobiography, which commences with the fall of Adam.

In the consideration of his second proposition, Dr. Leigh is a little obscure. He confounds his arranged and classified facts with the results of reasoning upon them, and labors hard to show that these results have the same force with the facts themselves. Now this is utterly opposed not only to the whole tenor of modern science, but to the rules of the Novum Organon itself. Admit this for one moment and the whole power of the ***negative instance*** is gone, and that just and righteous dictator of science forever deposed.

It is precisely here that the mediæval philosophers so grossly erred. It was because they attached to the authority of Aristotle the same or even more importance than to the evidence of their own senses, that ignorance and superstition in science and slavery to a master's dictum so long prevailed. The true natural philosopher must be

"Nullius addictus jurare in verba magistri,"

nor even on his own. He must be ready to abandon any theory, however brilliant, when the broad light of truth shows him that is only tinsel and not pure gold.

Dr. Leigh cites the examples of Newton and Gœthe, the one of whom saw gravitation in a falling apple, and the other detected the vertebral conformation in the skull of a deer. Now with all due deference to public opinion, we must say that we do not believe that Newton learned the law of gravitation from that apple. That its fall suggested to his mind the notions that masses were impelled to one another by some invisible power, is no doubt true. Like a true philosopher, he immediately proceeded to submit this idea to the test of facts. He examined the various phenomena of nature, and he found that every where the same thing took place, so that the law of gravitation became established.

As for the other illustration, we think it rather unfortunate. The vertebra theory, as it stands at present, in spite of the immense amount of research that has been lavished upon it, can scarcely be regarded as any thing else than a scientific phantasy, a sort of anatomical "Kubla Khan." It is now only a mode of seeing certain facts, only an imaginative expression of dimly seen harmonies of structure. So too the typical vertebra is a mere homological fiction, except so far as it exists in a pelican's neck, and then it is no more like the diagram, with its centron, and its neurapophyses and hæmapophyses, than the portraits of General Taylor which appeared during the Mexican war were like the redoubtable old soldier they pretended to represent. Science could very well dispense with all such fictions. They are as unreal as the figures the astronomer makes in the heavens, and certainly not more useful.

But it is under "thirdly" that the Dr. comes out particularly strong. What is to become of our general ideas, our theories, our general principles? he asks. We cannot see that they are at all affected by Dr. Bartlett's dictum.

What is a law of nature? Not as Dr. Leigh would have us believe, a general truth deduced from particular facts. It is nothing in the world but an expression of our own way of seeing a multitude of facts. Our minds are imperfect. They cannot grasp an uncounted multitude of disconnected facts and appreciate all their relations. They must toil laboriously, adding fact to fact, till they have acquired a sort of general expression for them all. Thus when we say coleoptera, we have a general idea of certain forms which are common to 80,000 species of insects. Now, though we call this expression a phrase of classification, our laws of nature, when closely examined, will be found to be identically the same, or rather the same mental act is performed when we discover a law of nature as when we establish a principle of classification. In both cases we only find a formula for a set of facts. We ascertain that the mysterious $x$ equals $a + b$. We know, indeed, very little of *a*, and hardly any thing of *b*, yet we see enough to know that it is a safe expression for the present state of knowledge. But, if at any future time, we should discover certain facts which would compel us to add *c* to our formula, our law would be revoked, or to speak more in accordance with our immeasurable ignorance, our formula would be overthrown and proved to be fallacious.

Take an example of the law of gravitation. What is it but an expression of a series of isolated facts daily occurring around us? That a stone falls to the ground, and that planets are held in their places by virtue of some invisible and incomprehensible power, and that this power is found to possess certain relations to time and space, this, not to go into minutiæ, is what we call the *law* of gravitation. But is it, after all, any thing else than a general expression of our own observed facts and their observed relations. The abstract reasoning on gravitation, a process, which Dr. Leigh so much admires, is precisely that part of the theory which is totally worthless and which has been rejected by science. The *expression of facts* which we call the law, is all that has been retained. The speculations about impulse, and all that sort of thing, by which gravitation was attempted to be accounted for, have shared the fate of the doctrines of mediæval physiology and the dreams of alchemy.

There is another class of general ideas in science, which serve as a sort of scaffolding to build the house, or rather as the assumptions in an arithmetical or algebraical problem, which guide the mind in a particular channel, and are to be confirmed, rejected or modified by the progress of investigation. These are the hypotheses or temporary theories of science, such, for example, as our present notions of the uses of fat in the economy, its relations to nutrition and its connection with respiration. Now, it is not to be denied, that the observed facts in regard to these points are numerous, and that they have shaped themselves in some minds, into a definite, consistent and beautiful theory, which will represent one of Dr. Leigh's *general ideas.* It is equally impossible, however, to deny that this theory has been constructed by the *assumption* of certain connecting facts, as the masses of the northern part of North America, figured in maps, are constructed by the assumption of the continuity of certain scattered tracts of land.

Now such theories as this are admitted into the body of science with caution and reserve. They are taken as assumptions to be hereafter worked out by subsequent observations, and only constitute a part of science, as the assumptions in the problem alluded to may be regarded as a part of the conclusion. If they are established, then they are received, but as they can be established only by *facts,* they then are really, what Dr. Bartlett calls them, an expression of facts and their observed relationships. It is certainly to this rigorous rule of Dr. Bartlett's that all medical science is now tending. Whatever has been *observed* or substantiated by observation, is received and nothing else. Hypotheses are taken at their true value, and estimated according to their probability, but are not by any sound reasoners, accepted as a part of the science, till they prove themselves by facts, that is, till they come to be the expression of observed facts and relationships.

This is precisely what makes the difference between physical and metaphysical science at the present time. The former, from its rigid adherence to facts, is daily becoming firmer and firmer. It has a solid basis to stand upon, and while it builds, it also demolishes all flimsy and unsubstantial structures, which have been added to the temple, no matter at what cost. As soon as they are found to be worthless they are thrown down, and their place is filled by more durable matter. Metaphysical science, on the other hand, being a mixture of fact and speculation, which are so artfully interwoven as to be inseparable, and each of which is of equal authority, is constantly shifting, and its more judicious students feel that what is added is equally doubtful with what is torn down. It is emphatically a science of opinions, and its history is only a record of the various ways in which men have viewed their own mental operations and the various deductions at which different philosophers have arrived, when starting from the same premises.

Being firmly convinced that, were Dr. Leigh's principles to be carried out to their full extent they would lead to results, undoubtedly not contemplated by himself, we have entered this friendly protest against them, and with great respect for his abilities, we are nevertheless constrained to differ from his conclusions.

